# *N*-(2-Hy­droxy-1,1-dimethyl­eth­yl)-4-methyl­benzene­sulfonamide

**DOI:** 10.1107/S1600536811000614

**Published:** 2011-01-08

**Authors:** Aziz-ur-Rehman, Amina Yasin, Mehmet Akkurt, Nadia Abbas, Muhammad Athar Abbasi, Islam Ullah Khan

**Affiliations:** a Department of Chemistry, Government College University, Lahore 54000, Pakistan; b Department of Physics, Faculty of Sciences, Erciyes University, 38039 Kayseri, Turkey

## Abstract

In the title mol­ecule, C_11_H_17_NO_3_S, the S atom has a distorted tetra­hedral geometry [maximum deviation: O—S—O = 119.08 (9)°]. In the crystal, mol­ecules are connected by inter­molecular N—H⋯O, O—H⋯O and C—H⋯O hydrogen bonds, forming layers of mol­ecules aligned parallel to (110). The 2-methyl­propan-1-ol group of the mol­ecule is disordered over two positions with an 0.592 (4):0.408 (4) occupancy ratio.

## Related literature

For background to the biological activity of sulfonamide derivatives, see: Berredjem *et al.* (2000 ▶); Lee & Lee (2002 ▶); Soledade *et al.* (2006 ▶); Xiao & Timberlake (2000 ▶). For some of our structural studies on various sulfonamide derivatives, see: Asiri *et al.* (2009 ▶); Aziz-ur-Rehman *et al.* (2010*a* ▶,*b* ▶).
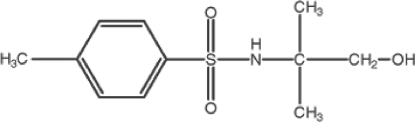


## Experimental

### 

#### Crystal data

C_11_H_17_NO_3_S
*M* *_r_* = 243.33
Monoclinic, 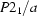

*a* = 10.4870 (3) Å
*b* = 9.0760 (3) Å
*c* = 13.4930 (5) Å
β = 97.755 (2)°
*V* = 1272.52 (7) Å^3^
*Z* = 4
Mo *K*α radiation
μ = 0.25 mm^−1^
*T* = 296 K
0.27 × 0.16 × 0.11 mm

#### Data collection

Bruker APEXII CCD diffractometer
11733 measured reflections
3116 independent reflections
2361 reflections with *I* > 2σ(*I*)
*R*_int_ = 0.029

#### Refinement

*R*[*F* ^2^ > 2σ(*F* ^2^)] = 0.045
*wR*(*F* ^2^) = 0.135
*S* = 1.01
3116 reflections
184 parameters
8 restraints
H-atom parameters constrained
Δρ_max_ = 0.37 e Å^−3^
Δρ_min_ = −0.31 e Å^−3^

### 

Data collection: *APEX2* (Bruker, 2007 ▶); cell refinement: *SAINT* (Bruker, 2007 ▶); data reduction: *SAINT*; program(s) used to solve structure: *SHELXS97* (Sheldrick, 2008 ▶); program(s) used to refine structure: *SHELXL97* (Sheldrick, 2008 ▶); molecular graphics: *ORTEP-3 for Windows* (Farrugia, 1997 ▶); software used to prepare material for publication: *WinGX* (Farrugia, 1999 ▶) and *PLATON* (Spek, 2009 ▶).

## Supplementary Material

Crystal structure: contains datablocks global, I. DOI: 10.1107/S1600536811000614/sj5093sup1.cif

Structure factors: contains datablocks I. DOI: 10.1107/S1600536811000614/sj5093Isup2.hkl

Additional supplementary materials:  crystallographic information; 3D view; checkCIF report

## Figures and Tables

**Table 1:** Hydrogen-bond geometry (Å, °)

*D*—H⋯*A*	*D*—H	H⋯*A*	*D*⋯*A*	*D*—H⋯*A*
N1—H1⋯O3*A*^i^	0.86	2.23	2.888 (10)	133
O3*A*—H3*A*⋯O1^ii^	0.82	2.15	2.894 (12)	151
C10*A*—H10*B*⋯O2^iii^	0.96	2.59	3.488 (4)	155

Symmetry codes: (i) 
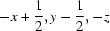
; (ii) 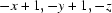; (iii) 
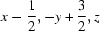
.

**Fig. 1.**
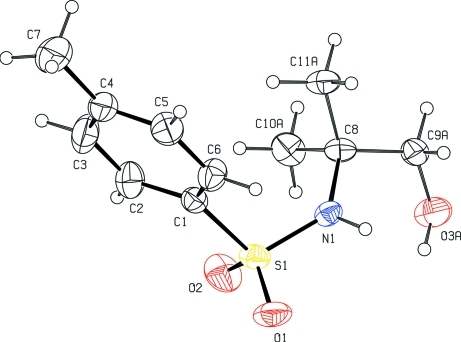
View of the major component of the disordered molecule shown with 30% probability displacement ellipsoids.

**Fig. 2.**
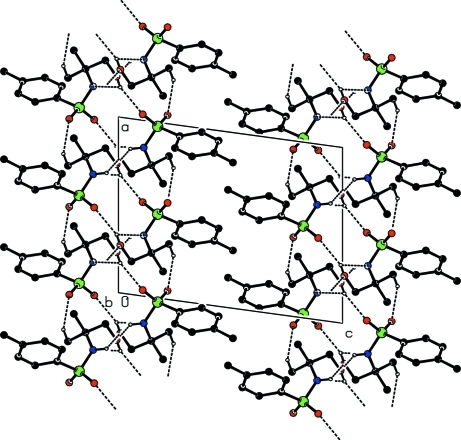
View of the packing and hydrogen bonding of (I) down the b axis. H atoms not involved in hydrogen bonding have been omitted for clarity and only the major component of the disorder is shown.

## supplementary crystallographic information

### Comment

Sulfonamide is an important functionality found in a number of synthetic as well as natural compounds and exhibiting various types of biological activity *e.g.* herbicidal, anti-malarial, anti-convulsant and anti-hypertensive (Soledade *et al.*, 2006; Xiao & Timberlake, 2000; Berredjem *et al.*, 2000; Lee & Lee, 2002) activities. As a contribution to a structural study of sulfonamide derivatives (Asiri *et al.*, 2009; Aziz-ur-Rehman *et al.*, 2010*a*,*b*;), we report here the title compound, *N*-(2-hydroxy-1,1-dimethylethyl)-4-methylbenzenesulfonamide, (I).

In the title molecule (I), (Fig. 1), the S atom has a distorted tetrahedral geometry [maximum deviation: O1—S1—O2 = 119.08 (9)°]. The molecule is twisted at the S atom, with a C1—S1—N1—C8 torsion angle of 68.22 (15)°.

The crystal packing is stabilized by intermolecular N—H···O and C—H···O hydrogen bonds, forming layers of molecules aligned parallel to the (110) lattice plane. (Table 1, Fig. 2).

### Experimental

A mixture of 4-methyl benzene sulfonyl chloride (10.0 mmoles; 1.90 g), 2-amino-2-methyl propanol (10.0 mmoles; 0.95 ml), aqueous sodium carbonate (10%; 10.0 ml) and water (25 ml) was stirred for half an hour at room temperature. The crude mixture was washed with water and dried. Product was dissolved in methanol and crystallized by slow evaporation of the solvent. Yield 76%.

### Refinement

All H atoms were positioned geometrically and allowed to ride on their parent atoms, with N—H = 0.86, O—H = 0.82 and C—H = 0.93–0.97 Å and allowed to ride on their parent atoms, with *U*_iso_(H) = 1.2*U*_eq_(C,*N*) for CH, CH_2_ and NH groups and 1.5*U*_eq_(C,*O*) for OH and CH_3_ groups. Disorder was observed with the 2-methylpropan-1-ol group of the title molecule, which was modeled in two positions with with an occupancy ratio of 0.592 (4):0.408 (4).

### Crystal data

**Table:**

C_11_H_17_NO_3_S	*F*(000) = 520
*M**_r_* = 243.33	*D*_x_ = 1.270 Mg m^−^^3^
Monoclinic, *P*2_1_/*a*	Mo *K*α radiation, λ = 0.71073 Å
Hall symbol: -P 2yab	Cell parameters from 3805 reflections
*a* = 10.4870 (3) Å	θ = 2.7–28.1°
*b* = 9.0760 (3) Å	µ = 0.25 mm^−^^1^
*c* = 13.4930 (5) Å	*T* = 296 K
β = 97.755 (2)°	Needle, colourless
*V* = 1272.52 (7) Å^3^	0.27 × 0.16 × 0.11 mm
*Z* = 4	

### Data collection

**Table:**

Bruker APEXII CCD diffractometer	2361 reflections with *I* > 2σ(*I*)
Radiation source: sealed tube	*R*_int_ = 0.029
graphite	θ_max_ = 28.3°, θ_min_ = 3.2°
φ and ω scans	*h* = −13→13
11733 measured reflections	*k* = −11→12
3116 independent reflections	*l* = −18→17

### Refinement

**Table:**

Refinement on *F*^2^	Primary atom site location: structure-invariant direct methods
Least-squares matrix: full	Secondary atom site location: difference Fourier map
*R*[*F*^2^ > 2σ(*F*^2^)] = 0.045	Hydrogen site location: inferred from neighbouring sites
*wR*(*F*^2^) = 0.135	H-atom parameters constrained
*S* = 1.01	*w* = 1/[σ^2^(*F*_o_^2^) + (0.0748*P*)^2^ + 0.2304*P*] where *P* = (*F*_o_^2^ + 2*F*_c_^2^)/3
3116 reflections	(Δ/σ)_max_ < 0.001
184 parameters	Δρ_max_ = 0.37 e Å^−^^3^
8 restraints	Δρ_min_ = −0.31 e Å^−^^3^

### Special details

**Table:**

Geometry. Bond distances, angles *etc*. have been calculated using the rounded fractional coordinates. All su's are estimated from the variances of the (full) variance-covariance matrix. The cell e.s.d.'s are taken into account in the estimation of distances, angles and torsion angles
Refinement. Refinement on *F*^2^ for ALL reflections except those flagged by the user for potential systematic errors. Weighted *R*-factors *wR* and all goodnesses of fit *S* are based on *F*^2^, conventional *R*-factors *R* are based on *F*, with *F* set to zero for negative *F*^2^. The observed criterion of *F*^2^ > σ(*F*^2^) is used only for calculating -*R*-factor-obs *etc*. and is not relevant to the choice of reflections for refinement. *R*-factors based on *F*^2^ are statistically about twice as large as those based on *F*, and *R*-factors based on ALL data will be even larger.

### Fractional atomic coordinates and isotropic or equivalent isotropic displacement parameters (Å^2^)

**Table:**

	*x*	*y*	*z*	*U*_iso_*/*U*_eq_	Occ. (<1)
S1	0.47716 (4)	0.43875 (5)	0.17517 (3)	0.0489 (2)	
O1	0.53388 (14)	0.34471 (17)	0.10820 (12)	0.0738 (6)	
O2	0.54916 (15)	0.56058 (18)	0.21936 (14)	0.0805 (6)	
O3A	0.2711 (12)	0.7568 (11)	0.0084 (7)	0.086 (3)	0.592 (4)
N1	0.34572 (13)	0.49570 (15)	0.11298 (10)	0.0417 (4)	
C1	0.43891 (15)	0.32645 (19)	0.27376 (12)	0.0430 (5)	
C2	0.4783 (2)	0.3647 (2)	0.37187 (15)	0.0639 (7)	
C3	0.4433 (3)	0.2773 (3)	0.44705 (16)	0.0779 (9)	
C4	0.3693 (2)	0.1524 (3)	0.42712 (16)	0.0672 (7)	
C5	0.3328 (2)	0.1157 (3)	0.32844 (17)	0.0669 (7)	
C6	0.36707 (19)	0.2008 (2)	0.25219 (14)	0.0571 (6)	
C7	0.3293 (3)	0.0610 (4)	0.5110 (2)	0.1129 (14)	
C8	0.25142 (16)	0.6009 (2)	0.14690 (13)	0.0469 (5)	
C9A	0.1880 (3)	0.6689 (4)	0.0581 (2)	0.0521 (10)	0.592 (4)
C10A	0.3165 (3)	0.7094 (4)	0.2253 (3)	0.0617 (11)	0.592 (4)
C11A	0.1552 (3)	0.5071 (4)	0.1991 (3)	0.0551 (11)	0.592 (4)
C9B	0.2902 (5)	0.7615 (5)	0.1091 (4)	0.0577 (16)	0.408 (4)
C10B	0.1167 (5)	0.5723 (7)	0.0779 (5)	0.078 (2)	0.408 (4)
O3B	0.2923 (17)	0.7726 (13)	0.0068 (8)	0.060 (3)	0.408 (4)
C11B	0.2318 (7)	0.6089 (7)	0.2491 (4)	0.078 (2)	0.408 (4)
H1	0.32740	0.46180	0.05320	0.0500*	
H9A1	0.15060	0.59280	0.01270	0.0620*	0.592 (4)
H5	0.28380	0.03130	0.31310	0.0800*	
H6	0.34180	0.17360	0.18600	0.0680*	
H7A	0.38440	0.08280	0.57200	0.1690*	
H7B	0.33600	−0.04170	0.49540	0.1690*	
H7C	0.24180	0.08370	0.51900	0.1690*	
H10A	0.35700	0.65560	0.28230	0.0920*	0.592 (4)
H10B	0.25290	0.77470	0.24570	0.0920*	0.592 (4)
H10C	0.38010	0.76560	0.19680	0.0920*	0.592 (4)
H11A	0.19980	0.46320	0.25860	0.0830*	0.592 (4)
H11B	0.11900	0.43110	0.15440	0.0830*	0.592 (4)
H11C	0.08760	0.56940	0.21640	0.0830*	0.592 (4)
H2	0.52800	0.44860	0.38710	0.0770*	
H9A2	0.11830	0.72980	0.07540	0.0620*	0.592 (4)
H3	0.47040	0.30320	0.51320	0.0940*	
H3A	0.32910	0.70550	−0.00800	0.1290*	0.592 (4)
H9B1	0.22970	0.83320	0.12860	0.0690*	0.408 (4)
H3B	0.34680	0.71640	−0.00990	0.0900*	0.408 (4)
H9B2	0.37480	0.78700	0.14320	0.0690*	0.408 (4)
H10D	0.13040	0.56780	0.00910	0.1180*	0.408 (4)
H10E	0.05830	0.65120	0.08680	0.1180*	0.408 (4)
H10F	0.08080	0.48070	0.09670	0.1180*	0.408 (4)
H11D	0.20820	0.51350	0.27130	0.1180*	0.408 (4)
H11E	0.16430	0.67800	0.25610	0.1180*	0.408 (4)
H11F	0.30980	0.64070	0.28890	0.1180*	0.408 (4)

### Atomic displacement parameters (Å^2^)

**Table:**

	*U*^11^	*U*^22^	*U*^33^	*U*^12^	*U*^13^	*U*^23^
S1	0.0392 (2)	0.0492 (3)	0.0606 (3)	−0.0009 (2)	0.0154 (2)	0.0076 (2)
O1	0.0678 (9)	0.0802 (10)	0.0825 (10)	0.0284 (8)	0.0431 (8)	0.0209 (8)
O2	0.0622 (9)	0.0712 (10)	0.1048 (13)	−0.0276 (7)	−0.0005 (8)	0.0140 (9)
O3A	0.084 (4)	0.093 (5)	0.089 (4)	0.048 (4)	0.042 (3)	0.051 (3)
N1	0.0492 (7)	0.0395 (7)	0.0386 (7)	0.0052 (6)	0.0139 (5)	0.0000 (6)
C1	0.0384 (7)	0.0445 (9)	0.0460 (8)	0.0031 (6)	0.0056 (6)	0.0023 (7)
C2	0.0726 (12)	0.0601 (12)	0.0549 (11)	−0.0001 (10)	−0.0058 (9)	−0.0051 (9)
C3	0.0952 (17)	0.0927 (18)	0.0427 (10)	0.0160 (15)	−0.0024 (10)	0.0014 (11)
C4	0.0581 (11)	0.0864 (16)	0.0589 (11)	0.0148 (11)	0.0144 (9)	0.0258 (11)
C5	0.0592 (11)	0.0678 (13)	0.0730 (13)	−0.0125 (10)	0.0064 (10)	0.0181 (11)
C6	0.0628 (11)	0.0592 (11)	0.0482 (9)	−0.0139 (9)	0.0038 (8)	0.0030 (8)
C7	0.100 (2)	0.155 (3)	0.0892 (19)	0.022 (2)	0.0323 (17)	0.066 (2)
C8	0.0437 (8)	0.0455 (9)	0.0548 (10)	0.0037 (7)	0.0184 (7)	−0.0045 (7)
C9A	0.0461 (16)	0.0575 (19)	0.0534 (16)	0.0148 (14)	0.0098 (12)	0.0115 (14)
C10A	0.0620 (19)	0.0518 (19)	0.070 (2)	0.0075 (15)	0.0041 (16)	−0.0229 (16)
C11A	0.0508 (17)	0.0559 (19)	0.064 (2)	0.0055 (14)	0.0272 (14)	0.0030 (15)
C9B	0.067 (3)	0.043 (2)	0.067 (3)	0.011 (2)	0.023 (2)	0.005 (2)
C10B	0.044 (3)	0.084 (4)	0.106 (5)	0.012 (3)	0.007 (3)	0.012 (3)
O3B	0.086 (6)	0.044 (3)	0.056 (4)	0.021 (3)	0.034 (3)	0.010 (3)
C11B	0.103 (5)	0.078 (4)	0.063 (3)	0.039 (4)	0.044 (3)	0.018 (3)

### Geometric parameters (Å, °)

**Table:**

S1—O1	1.4297 (16)	C2—H2	0.9300
S1—O2	1.4237 (17)	C3—H3	0.9300
S1—N1	1.5997 (14)	C5—H5	0.9300
S1—C1	1.7645 (17)	C6—H6	0.9300
O3A—C9A	1.415 (12)	C7—H7A	0.9600
O3B—C9B	1.387 (12)	C7—H7C	0.9600
O3A—H3A	0.8200	C7—H7B	0.9600
O3B—H3B	0.8200	C9A—H9A1	0.9700
N1—C8	1.490 (2)	C9A—H9A2	0.9700
N1—H1	0.8600	C9B—H9B2	0.9700
C1—C2	1.377 (3)	C9B—H9B1	0.9700
C1—C6	1.376 (3)	C10A—H10C	0.9600
C2—C3	1.376 (3)	C10A—H10A	0.9600
C3—C4	1.380 (4)	C10A—H10B	0.9600
C4—C5	1.376 (3)	C10B—H10E	0.9600
C4—C7	1.508 (4)	C10B—H10F	0.9600
C5—C6	1.373 (3)	C10B—H10D	0.9600
C8—C10B	1.605 (6)	C11A—H11C	0.9600
C8—C11B	1.423 (6)	C11A—H11A	0.9600
C8—C9B	1.614 (5)	C11A—H11B	0.9600
C8—C9A	1.430 (3)	C11B—H11D	0.9600
C8—C10A	1.536 (4)	C11B—H11E	0.9600
C8—C11A	1.559 (4)	C11B—H11F	0.9600
			
S1···H10A	2.8400	H1···H3A	2.3600
S1···H10C	3.1600	H1···C9B^i^	2.9800
S1···H11F	3.0900	H9A1···H1	2.2100
O1···C9A^i^	3.404 (3)	H9A1···H11B	2.4700
O1···O3A^ii^	2.894 (12)	H2···O2	2.5200
O1···O3B^ii^	2.762 (15)	H9A2···H11C	2.4500
O2···C10A	2.799 (4)	H9A2···O2^ix^	2.8800
O3A···N1	2.815 (10)	H9A2···H10B	2.5600
O3A···N1^iii^	2.888 (10)	H3···H7A	2.3700
O3A···O1^ii^	2.894 (12)	H9B1···H10E	2.4500
O3B···O1^ii^	2.762 (15)	H9B1···H11E	2.3900
O3B···N1^iii^	2.859 (13)	H9B1···O2^ix^	2.5800
O3B···C10B^iii^	3.148 (14)	H3A···N1	2.5000
O3B···N1	2.910 (12)	H3A···H1	2.3600
O1···H3B^ii^	2.0200	H3A···O1^ii^	2.1500
O1···H3A^ii^	2.1500	H3B···N1	2.6000
O1···H6	2.8500	H3B···H1	2.4800
O1···H11B^iv^	2.7000	H3B···O1^ii^	2.0200
O2···H2	2.5200	H9B2···O2	2.8500
O2···H10C	2.5600	H9B2···H11F	2.5400
O2···H11E^v^	2.6800	H9B2···H10E^v^	2.2300
O2···H9B2	2.8500	H9B2···C10B^v^	3.0700
O2···H11F	2.8900	H5···H10B^x^	2.5100
O2···H5^iv^	2.7400	H5···O2^xi^	2.7400
O2···H10B^v^	2.5900	H5···H7B	2.5300
O2···H10A	2.4500	H6···O3A^i^	2.8300
O2···H9A2^v^	2.8800	H6···O1	2.8500
O2···H9B1^v^	2.5800	H7A···H3	2.3700
O3A···H10C	2.6400	H7A···H11E^vi^	2.5900
O3A···H6^iii^	2.8300	H7A···C11B^vi^	2.8600
O3A···H1	2.7900	H7A···H11D^vi^	2.5200
O3A···H1^iii^	2.2300	H7B···H5	2.5300
O3B···H10D^iii^	2.8200	H10A···S1	2.8400
O3B···H10D	2.5200	H10A···H11A	2.3900
O3B···H1^iii^	2.2100	H10A···O2	2.4500
O3B···H10F^iii^	2.7900	H10B···H9A2	2.5600
O3B···H1	2.9000	H10B···O2^ix^	2.5900
N1···O3B	2.910 (12)	H10B···H5^xii^	2.5100
N1···O3A	2.815 (10)	H10B···H11C	2.5400
N1···O3B^i^	2.859 (13)	H10C···S1	3.1600
N1···O3A^i^	2.888 (10)	H10C···O3A	2.6400
N1···H3A	2.5000	H10C···O2	2.5600
N1···H3B	2.6000	H10D···O3B	2.5200
C1···C11B	3.347 (7)	H10D···H1	2.2800
C1···C11A	3.427 (4)	H10D···O3B^i^	2.8200
C6···C11A	3.571 (4)	H10D···C10B^vii^	2.9800
C7···C11B^vi^	3.411 (6)	H10D···H10F^vii^	2.5100
C9A···O1^iii^	3.404 (3)	H10E···H9B1	2.4500
C10A···O2	2.799 (4)	H10E···H11E	2.4100
C10B···C10B^vii^	3.278 (9)	H10E···C9B^ix^	2.9800
C10B···O3B^i^	3.148 (14)	H10E···H9B2^ix^	2.2300
C11A···C6	3.571 (4)	H10F···O3B^i^	2.7900
C11A···C1	3.427 (4)	H10F···C10B^vii^	2.9600
C11B···C1	3.347 (7)	H10F···H10D^vii^	2.5100
C11B···C7^viii^	3.411 (6)	H10F···H11D	2.5600
C1···H11D	2.9500	H11A···H10A	2.3900
C1···H11A	2.7800	H11A···C1	2.7800
C6···H11A	2.9700	H11A···C6	2.9700
C7···H11D^vi^	3.0500	H11B···O1^xi^	2.7000
C9A···H1^iii^	3.0500	H11B···H9A1	2.4700
C9B···H10E^v^	2.9800	H11C···H9A2	2.4500
C9B···H1^iii^	2.9800	H11C···H10B	2.5400
C10B···H10D^vii^	2.9800	H11D···C1	2.9500
C10B···H10F^vii^	2.9600	H11D···H10F	2.5600
C10B···H9B2^ix^	3.0700	H11D···C7^viii^	3.0500
C11B···H7A^viii^	2.8600	H11D···H7A^viii^	2.5200
H1···H3B	2.4800	H11E···H9B1	2.3900
H1···H10D	2.2800	H11E···H10E	2.4100
H1···O3B	2.9000	H11E···H7A^viii^	2.5900
H1···C9A^i^	3.0500	H11E···O2^ix^	2.6800
H1···O3B^i^	2.2100	H11F···S1	3.0900
H1···O3A^i^	2.2300	H11F···O2	2.8900
H1···O3A	2.7900	H11F···H9B2	2.5400
H1···H9A1	2.2100		
			
O1—S1—O2	119.08 (9)	C1—C6—H6	120.00
O1—S1—N1	105.30 (8)	C4—C7—H7A	110.00
O1—S1—C1	106.74 (9)	C4—C7—H7C	109.00
O2—S1—N1	109.85 (9)	H7A—C7—H7B	110.00
O2—S1—C1	107.13 (9)	H7A—C7—H7C	109.00
N1—S1—C1	108.34 (7)	H7B—C7—H7C	109.00
C9A—O3A—H3A	110.00	C4—C7—H7B	109.00
C9B—O3B—H3B	109.00	O3A—C9A—H9A1	109.00
S1—N1—C8	127.33 (11)	O3A—C9A—H9A2	109.00
S1—N1—H1	116.00	C8—C9A—H9A2	109.00
C8—N1—H1	116.00	H9A1—C9A—H9A2	108.00
S1—C1—C2	120.66 (14)	C8—C9A—H9A1	109.00
S1—C1—C6	119.53 (13)	O3B—C9B—H9B1	109.00
C2—C1—C6	119.80 (16)	O3B—C9B—H9B2	109.00
C1—C2—C3	119.22 (19)	C8—C9B—H9B1	109.00
C2—C3—C4	121.9 (2)	C8—C9B—H9B2	109.00
C3—C4—C7	120.8 (2)	H9B1—C9B—H9B2	108.00
C5—C4—C7	121.6 (2)	H10A—C10A—H10B	109.00
C3—C4—C5	117.6 (2)	H10A—C10A—H10C	110.00
C4—C5—C6	121.5 (2)	C8—C10A—H10A	109.00
C1—C6—C5	119.94 (18)	C8—C10A—H10B	109.00
N1—C8—C9B	106.0 (2)	C8—C10A—H10C	109.00
N1—C8—C11B	121.3 (3)	H10B—C10A—H10C	110.00
C9A—C8—C10A	114.4 (2)	C8—C10B—H10D	109.00
N1—C8—C10B	106.7 (3)	H10D—C10B—H10E	110.00
C10A—C8—C11A	107.1 (2)	H10D—C10B—H10F	110.00
C9B—C8—C10B	101.7 (3)	H10E—C10B—H10F	109.00
C9B—C8—C11B	109.6 (3)	C8—C10B—H10E	109.00
C10B—C8—C11B	109.8 (4)	C8—C10B—H10F	109.00
N1—C8—C9A	105.79 (18)	H11B—C11A—H11C	109.00
N1—C8—C10A	111.75 (17)	H11A—C11A—H11B	110.00
C9A—C8—C11A	111.0 (2)	H11A—C11A—H11C	109.00
N1—C8—C11A	106.60 (18)	C8—C11A—H11A	109.00
O3A—C9A—C8	113.2 (5)	C8—C11A—H11B	109.00
O3B—C9B—C8	114.7 (6)	C8—C11A—H11C	109.00
C3—C2—H2	120.00	C8—C11B—H11D	109.00
C1—C2—H2	120.00	C8—C11B—H11E	109.00
C4—C3—H3	119.00	C8—C11B—H11F	109.00
C2—C3—H3	119.00	H11D—C11B—H11E	110.00
C4—C5—H5	119.00	H11D—C11B—H11F	109.00
C6—C5—H5	119.00	H11E—C11B—H11F	109.00
C5—C6—H6	120.00		
			
O1—S1—N1—C8	−177.87 (14)	C6—C1—C2—C3	−1.0 (3)
O2—S1—N1—C8	−48.49 (17)	S1—C1—C6—C5	−177.58 (16)
C1—S1—N1—C8	68.22 (15)	C2—C1—C6—C5	1.3 (3)
O1—S1—C1—C2	128.69 (16)	C1—C2—C3—C4	−0.3 (4)
O1—S1—C1—C6	−52.45 (17)	C2—C3—C4—C5	1.1 (4)
O2—S1—C1—C2	0.09 (18)	C2—C3—C4—C7	−178.3 (3)
O2—S1—C1—C6	178.96 (15)	C3—C4—C5—C6	−0.8 (4)
N1—S1—C1—C2	−118.36 (15)	C7—C4—C5—C6	178.6 (2)
N1—S1—C1—C6	60.50 (16)	C4—C5—C6—C1	−0.4 (3)
S1—N1—C8—C9A	153.47 (18)	N1—C8—C9A—O3A	−65.4 (5)
S1—N1—C8—C10A	28.3 (2)	C10A—C8—C9A—O3A	58.1 (5)
S1—N1—C8—C11A	−88.3 (2)	C11A—C8—C9A—O3A	179.4 (5)
S1—C1—C2—C3	177.89 (18)		

Symmetry codes: (i) −*x*+1/2, *y*−1/2, −*z*; (ii) −*x*+1, −*y*+1, −*z*; (iii) −*x*+1/2, *y*+1/2, −*z*; (iv) *x*+1/2, −*y*+1/2, *z*; (v) *x*+1/2, −*y*+3/2, *z*; (vi) −*x*+1/2, *y*−1/2, −*z*+1; (vii) −*x*, −*y*+1, −*z*; (viii) −*x*+1/2, *y*+1/2, −*z*+1; (ix) *x*−1/2, −*y*+3/2, *z*; (x) *x*, *y*−1, *z*; (xi) *x*−1/2, −*y*+1/2, *z*; (xii) *x*, *y*+1, *z*.

### Hydrogen-bond geometry (Å, °)

**Table:**

*D*—H···*A*	*D*—H	H···*A*	*D*···*A*	*D*—H···*A*
N1—H1···O3A^i^	0.86	2.23	2.888 (10)	133
O3A—H3A···N1	0.82	2.50	2.815 (10)	104
O3A—H3A···O1^ii^	0.82	2.15	2.894 (12)	151
C2—H2···O2	0.93	2.52	2.891 (3)	104
C10A—H10A···O2	0.96	2.45	2.799 (4)	101
C10A—H10B···O2^ix^	0.96	2.59	3.488 (4)	155

Symmetry codes: (i) −*x*+1/2, *y*−1/2, −*z*; (ii) −*x*+1, −*y*+1, −*z*; (ix) *x*−1/2, −*y*+3/2, *z*.
